# The association between the provision of recommendations for stillbirth prevention and the stillbirth reduction: a regional population-based study in Japan

**DOI:** 10.1186/s12889-026-27488-w

**Published:** 2026-04-23

**Authors:** Shigeki Koshida, Shinsuke Tokoro, Daisuke Katsura, Shunichiro Tsuji, Jun Matsubayashi, Takashi Murakami, Kentaro Takahashi

**Affiliations:** 1 Department of Perinatal Center, Shiga University of Medical Science, Tsukinowa-cho, Seta, Otsu, Shiga 520-2192 Japan; 2 Department of Obstetrics and Gynecology, Shiga University of Medical Science, Otsu, Shiga Japan; 3 Center for Clinical Research and Advanced Medicine, Shiga University of Medical Science, Otsu, Shiga Japan

**Keywords:** Stillbirth, Prevention, Recommendation, Intervention

## Abstract

**Background:**

Shiga Prefecture in Japan still had higher stillbirth rates than the national average before 2010. To improve stillbirth rates in Shiga, we have informed both obstetricians and pregnant women in our region of recommendations for stillbirth prevention after peer-reviewing all stillbirth cases in Shiga since 2013. We, therefore, evaluated the reduction in the stillbirth rate and preventable stillbirths in Shiga after the intervention.

**Methods:**

This was a population-based study in Shiga Prefecture, Japan. We assessed outcomes over 15 years divided into three 5-year periods: before- (2008-12), early- (2013-17), and late-intervention (2018-22) period. The primary outcome was the rate of stillbirth in Shiga, and the secondary outcome was the rate of preventable stillbirth in each period.

**Results:**

The stillbirth rate in Shiga significantly decreased by 40% in the late-intervention period compared to that in the before-intervention period (95% CI: (−52%, (−24%), and it also significantly decreased compared to the national average between those periods (rate ratio: 0.76, 95% CI: 0.61, 0.96). It improved to the second lowest level in Japan during the late-intervention period. The rate of possibly preventable stillbirths in the late-intervention was significantly lower than that in the before-intervention period.

**Conclusions:**

Informing healthcare providers and pregnant women of the recommendations for stillbirth prevention was associated with a reduction in stillbirths.

**Supplementary Information:**

The online version contains supplementary material available at 10.1186/s12889-026-27488-w.

## Introduction

 Stillbirth is defined as fetal death prior to the complete expulsion or extraction from the mother [1, 2]. Stillbirths, along with early neonatal demises, constitute perinatal mortality. The stillbirth rate is used as a marker of the quality of perinatal care among countries or regions. Owing to improvements in maternal care, the stillbirth rate in high-income countries has decreased rapidly over the past several decades [3]. However, although Japan has achieved the lowest stillbirth rate (≥ 28 weeks of gestation) among high-income countries at 1.58 per 1000 births in 2021 (vs. 2.09 in Norway, 2.22 in Italy, 2.39 in Australia, 2.73 in the US and UK and, 3.07 in France) [3, 4], the regional stillbirth rates vary more than 2-fold across Japanese prefectures [5].

Shiga Prefecture, located in the center of Japan, has the third highest birth rate in Japan with approximately 12,000 annual births, and had higher stillbirth rates than the Japanese average for the decade beginning in 2000. Quantitative and qualitative assessments are needed to clarify the perinatal problems in a region in order to improve regional perinatal care indicators. We therefore began collecting data on all stillbirths in the region and peer-reviewed it [6]. In addition, we compiled recommendations for preventing stillbirths and disseminated them to regional healthcare providers and pregnant women [7]. However, the impact of our interventions to prevent stillbirths on the perinatal indicators of the region remains unclear.

We therefore investigated the changes in the regional stillbirth rate following these interventions. We also evaluated the association between these interventions and stillbirth prevention.

## Methods

### Study design and ethical statement

This retrospective population-based study was performed in Shiga Prefecture, Japan, from 2008 to 2022. This study assessed the impact of disseminating recommendations for preventing stillbirths derived from a review of all stillbirth cases in Shiga.

This study was approved by the Institutional Review Board of Shiga University of Medical Science on June 12, 2019 (Approval No. R2017-151). Informed consent from participants with stillbirth was obtained in the form of opt-out on the website. The datasets analyzed in the current study on demographic trends are available from participants in the repository of the Ministry of Health, Labour and Welfare without requiring informed consent from participants [5]. This study was conducted in accordance with the principles of the Declaration of Helsinki.

### Reviewing stillbirth cases and intervention for stillbirth prevention

While stillbirth is legally defined in Japan as the delivery of a baby with no signs of life at or after 12 weeks of gestation, regardless of birth weight, the definition based on 22 weeks of gestation is more widely accepted in medical and epidemiological research [1, 2, 5]. This definition is also adopted in Japan’s vital statistics, which compile and publish stillbirth data based on fetal deaths occurring at or after 22 weeks of gestation [5]. It aligns with the World Health Organization definition, which defines stillbirth as fetal death at or after 22 weeks of gestation, and facilitates international comparability [1, 2]. Therefore, in this study, stillbirth was defined as fetal death at or after 22 weeks of gestation.

This definition did not change during the study period. Detailed quality checks are not routinely performed when reporting stillbirth certificates in Japan. As official stillbirth statistics lack detailed information on the circumstances leading to stillbirth, we conducted a comprehensive review of stillbirth cases to determine their causes and evaluate their preventability. Obstetric healthcare in Japan is categorized into three main facilities: primary obstetric clinics for low-risk pregnancies, general hospitals for middle-risk pregnancies, and perinatal centers for high-risk pregnancies.

We examined stillbirth cases since 2012 by investigating stillbirth certificates with permission from the Ministry of Health, Labour and Welfare (MHLW). We sent detailed questionnaires covering pregnancy history, laboratory results, and clinical findings to each facility that submitted a stillbirth certificate. A peer-review team of experienced obstetricians and neonatologists reviewed the returned questionnaires, evaluated the preventability of each case, and classified preventability as either possible or impossible. The team decided on preventability based on consensus, without specific definitions or criteria, although national obstetric guidelines available at the time were used as a reference in making these judgments [8]. The team did not base their judgments on the primary cause of stillbirth. In cases where consensus could not be achieved, the final decision was determined by majority vote.

The peer-review team simultaneously determined recommendations for preventing stillbirth in each case deemed preventable. For obstetricians, these recommendations included improving the diagnosis and perinatal management of mothers and fetuses in poor condition, and referring high-risk pregnancies earlier to higher-level perinatal centers [6]. For pregnant women and midwives, recommendations included early visits to healthcare providers if they perceive decreased fetal movement (DFM), supported by the promotion of fetal movement counting (FMC) in our region [7]. These recommendations were based on findings from our review of stillbirth cases. In multiple cases, high-risk pregnancies had not been referred to perinatal centers and were subsequently presumed to have contributed to fetal death [6]. This suggested that improvements in referral practices and perinatal management by obstetricians could be both feasible and effective in preventing stillbirth. Additionally, we frequently observed cases in which pregnant women had perceived DFM for several days but did not seek medical attention until a scheduled visit, at which point intrauterine fetal death was confirmed [7]. These patterns led us to introduce FMC as an intervention to encourage earlier consultation when DFM is perceived. Since 2013, we have shared these recommendations with obstetricians, midwives, public health nurses, and pregnant women in the region.

We defined the intervention in this study as the provision of recommendations for stillbirth prevention in the region (Table 1). For obstetricians, we communicated recommendations based on reviewed avoidable stillbirth cases directly to reporting facilities and shared them at the annual obstetricians’ conference in Shiga. For pregnant women, we created a fetal movement (FM) counting chart and provided it to each patient through an obstetric caregiver in the region. The women were asked to record the time taken to perceive 10 FMs on the chart once per day at any time from 34 weeks of gestation until delivery. They were also asked to report to their caregiver if it took more than 30 min to perceive 10 FMs. For midwives and public health nurses, we visited public health centers to convey recommendations to pregnant women, ensuring that the information reached them.

**Table 1:** Interventions for obstetricians and pregnant women

Target	**Obstetricians**		**Pregnant women**
	Individuals with avoidable stillbirths	Participating groups	
Type	Individual feedback	Seminar-based sharing	Self-monitoring of fetal movements
Mode	Directly to individuals from the peer-review team	In-person presentation by the peer-review team	Face-to-face instruction by midwives
Timing	After each peer-review	Annual meeting in our region	From 34 weeks of gestation to their delivery
Content	Feedback on problems in avoidable stillbirths and recommendations for prevention ・Earlier referral of high-risk pregnancies, including FGR, threatened premature labor (< 28w), MD twin pregnancy (≥16w), Chorioamnionitis (< 36w) to perinatal centers ・Improvement in the diagnosis of antenatal fetal anomalies, fetal heart rate monitoring, and FGR ・Improvement in neonatal resuscitation ・Instructions for pregnant women to promptly make contact when perceiving DFM		Provision of FM count chart and instruction for daily recording of the time taken to perceive 10 FMs on the chart ・Report to their caregiver if it takes more than 30 minutes for 10 FMs to occur

*FGR* Fetal growth restriction, *MD* Monochorionic diamniotic, *DFM* Decreased fetal movement, *FM* Fetal movement

### Data collection

In Japan, the MHLW collects birth and stillbirth data from the municipal offices. Birth certificates and stillbirth notifications are submitted by hospitals or individuals, and local governments report them to the MHLW. The Ministry aggregates and publishes these data, which are available for download as official statistical information [5].

There are approximately 10,000–13,000 annual births in Shiga Prefecture, with a population of 1.4 million [5]. We obtained the number of births and stillbirths after 22 weeks of gestation in each prefecture of Japan [5]. The stillbirth rate was defined as the number of stillbirths after 22 weeks of gestation per 1,000 births.

It should be noted that our definition of stillbirth differs from the legal Japanese definition, which includes fetal deaths occurring at or after 12 weeks of gestation. However, since the official database separately reports the number of stillbirths after 22 weeks, which are included in perinatal mortality statistics, we used these data without applying any special calculations [5].

### Outcome measures

Outcome measures were evaluated over a 15-year period from 2008 to 2022, divided into three 5-year periods: before-intervention (2008–2012), early-intervention (2013–2017), and late-intervention (2018–2022) periods. The primary outcome measure was the stillbirth rate in Shiga Prefecture during each period. The secondary outcome was the rate of stillbirths determined to have been prevented in each period. The stillbirth rate was defined as the number of stillbirths at or after 22 weeks of gestation divided 1,000 births.

### Statistical analyses

We calculated the total number of births and stillbirths in each period separately for Shiga Prefecture and Japan. We computed the birth rate as the number of births per 1,000 population and stillbirth rate as the number of stillbirths at or after 22 weeks of gestation per 1,000 births for each period, along with their 95% confidence intervals (CIs). We used Poisson regression to calculate the percentage change in the stillbirth rate and its 95% CI during the early- and late-intervention periods compared to the before-intervention period and compared the percentage change in stillbirth rate between Shiga Prefecture and Japan. We used Fisher’s exact test to compare the proportion of potentially avoidable stillbirth cases out of the total number of stillbirths during the early- and late-intervention periods with the before-intervention period.

All statistical analyses were performed using the IBM SPSS software program (ver. 23 IBM Japan, Tokyo, Japan) and SAS 9.4 (SAS Institute, Cary, NC, USA). Statistical significance was set at *P* < 0.05.

## Results

### Change in the stillbirth rate of Shiga Prefecture compared to the Japanese average

**Fig. 1:**
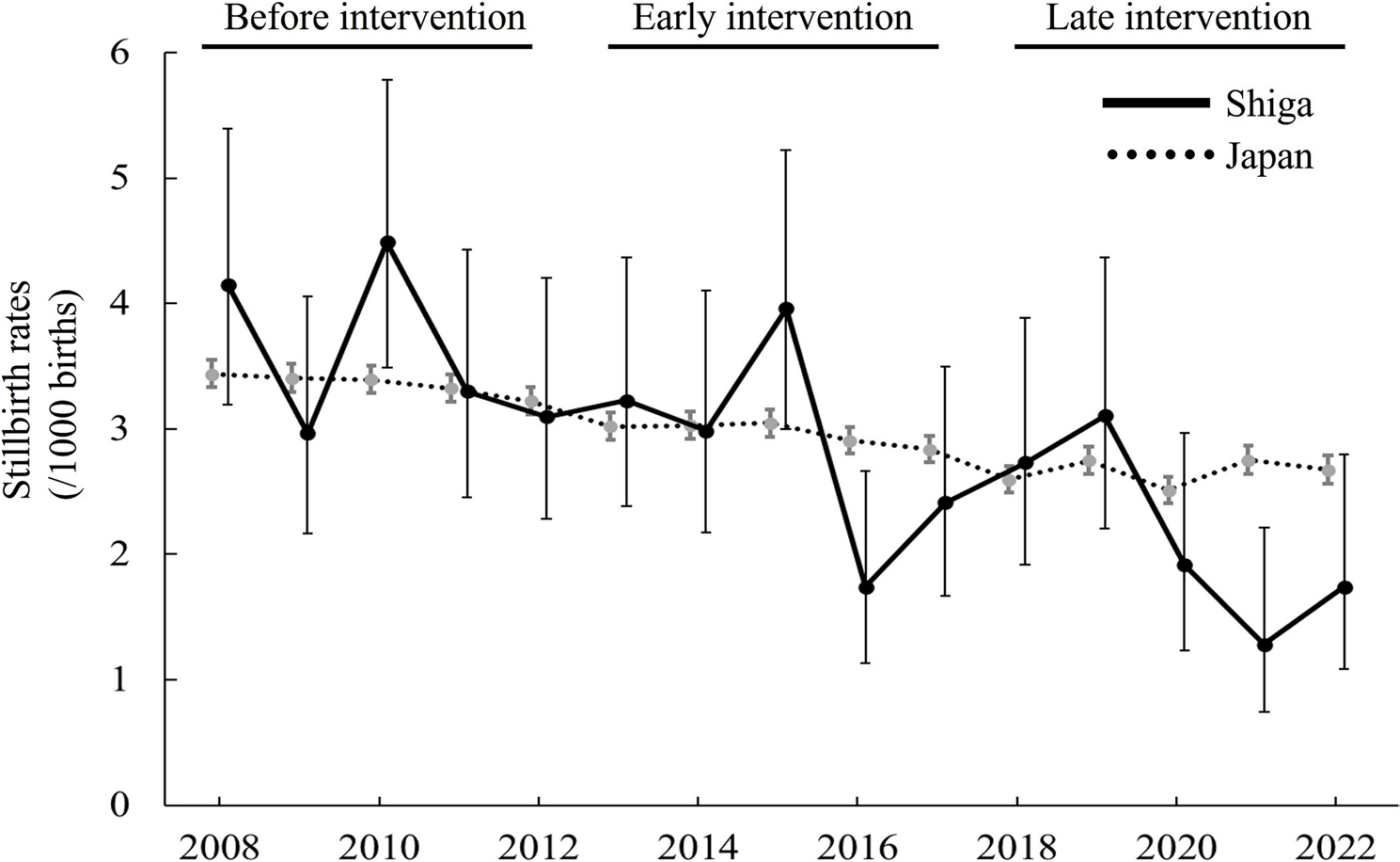
Stillbirth rates in Shiga Prefecture and Japan in the study period. The stillbirth rate was defined as the number of stillbirths ≥ 22 weeks of gestation per 1,000 births. Bold line: stillbirth rates in Shiga; dotted line: stillbirth rates in Japan. Error bars represent 95% confidence intervals for each annual estimate. Note: Stillbirth rates in Shiga were the lowest in Japan in 2016, 2021, and 2022

**Fig. 2:**
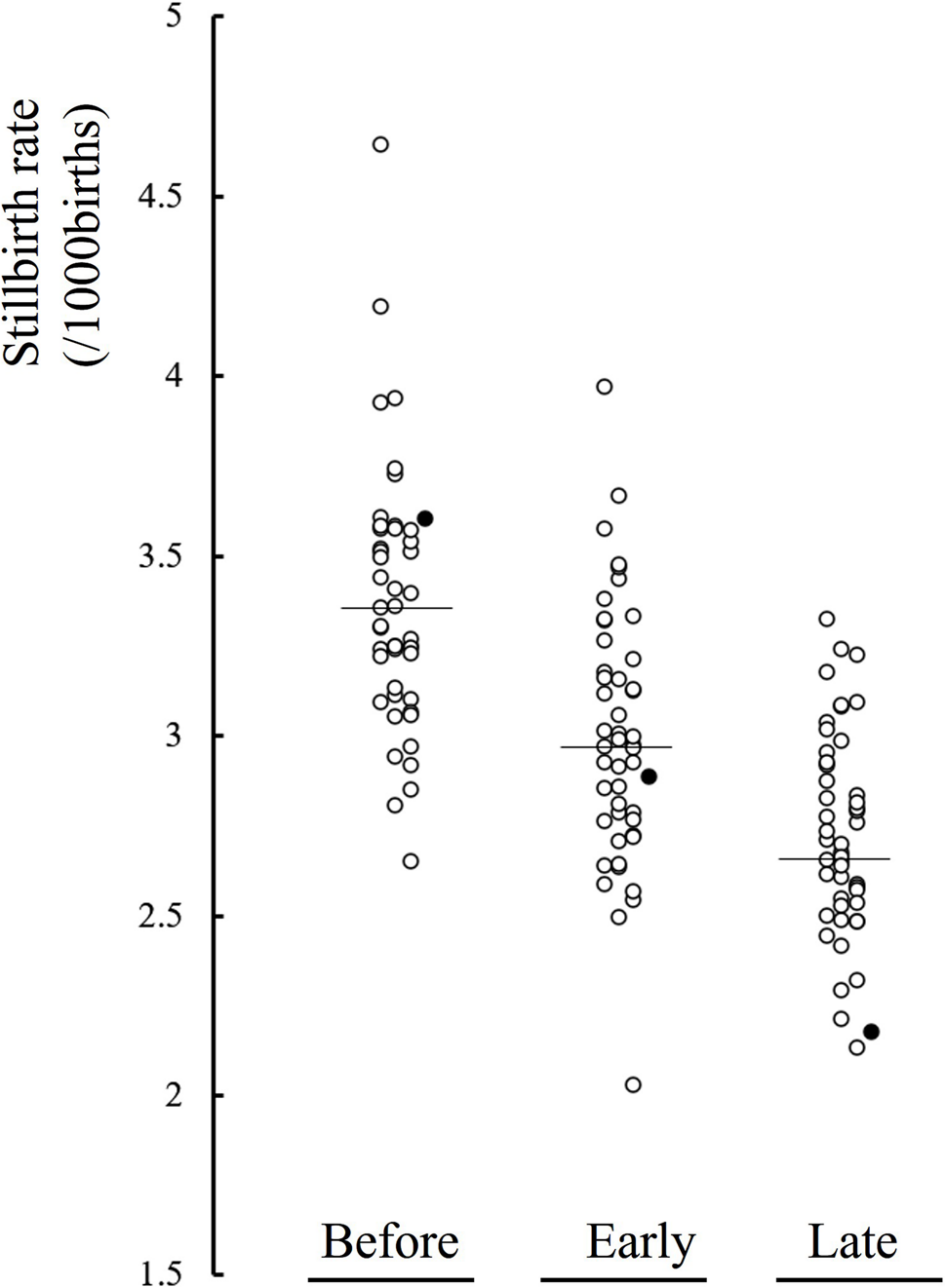
Average stillbirth rate in every prefecture of Japan in each study period. The circles (◯) indicate the average stillbirth rate during the study period in each prefecture in Japan, and the solid dots (●)　indicate the rate in Shiga. Horizontal lines represent the average stillbirth rate in Japan. The stillbirth rate was defined as the number of stillbirths ≥ 22 weeks of gestation per 1,000 births

The stillbirth rate in Japan decreased gradually across the study periods (3.44 per 1,000 live births in 2008 and 2.67 in 2022; Fig. 1). The stillbirth rate in Shiga Prefecture also decreased, but the rate of decrease accelerated during the second half of the study periods (Fig. 1). Notably, the stillbirth rate in Shiga Prefecture was the lowest (i.e. the best) among all 47 prefectures in 2016, 2021 and 2022 (1.74, 1.28, and 1.74, respectively). Examining the stillbirth rate by study period, the stillbirth rate in Shiga Prefecture was higher than the national average in the before-intervention period (3.6 in Shiga vs. 3.4 in Japan), about the same in the early-intervention period (2.9 vs. 3.0), and lower in the late-intervention period (2.2 vs. 2.7; Table 2). Shiga Prefecture had the 40th, 19th, and 2nd lowest stillbirth rates in Japan in the before-, early-, and late-intervention periods, respectively (Fig. 2).

**Table 2:** Perinatal background of Shiga prefecture and Japan during the study periods

	**Before (2008-2012)**		**Early (2013-2017)**		**Late (2018-2022)**	
	**Shiga**	**Japan**	**Shiga**	**Japan**	**Shiga**	**Japan**
Births						
number	66575	5320532	62036	4962075	52310	4206855
rates	9.6 [9.4–9.8]	8.4 [8.2–8.7]	8.9 [8.4–9.4]	7.9 [7.6–8.2]	7.6 [7.1–8.1]	6.8 [6.3–7.3]
Stillbirths						
number	240	17867	179	14735	114	11170
rates	3.6 [2.8–4.4]	3.4 [3.3–3.5]	2.9 [1.8–3.9]	3.0 [2.9–3.1]	2.2 [1.2–3.1]	2.7 [2.5–2.8]

Births rates are calculated per 1,000 population and stillbirth rates per 1,000 births for each study period

Both rates are presented as average [95% confidence intervals]

Table 3 shows the changes in stillbirth rates in Shiga Prefecture and Japan compared to the before-intervention period. In the early-intervention period, stillbirth rates in Shiga Prefecture and Japan changed by −20% (95% CI: −34%, −3%) and by −12% (−13%, −10%), respectively. The comparison of the percentage changes between Shiga Prefecture and Japan in the early-intervention period did not reach statistical significance (*p* = 0.32). In the late-intervention period, the stillbirth rates in Shiga Prefecture and Japan changed by −40% (95% CI: −52%, −24%) and −21% (−23%, −19%), respectively, compared with the before-intervention period. The difference in the percentage changes between Shiga Prefecture and Japan in the late-intervention period was statistically significant (*p* = 0.02).

**Table 3:** Changes in stillbirth rates in Shiga Prefecture and Japan after the intervention compared to before the intervention

	Shiga		Japan		Shiga/Japan	
	Percent Change, % (95% CI)	*P* value	Percent Change, % (95% CI)	*P* value	Rate ratio (95% CI)	*P* value
Early vs. before	−20 (−34 to −3)	0.02	−12 (−13 to −10)	< 0.001	0.91 (0.74 to 1.10)	0.32
Late vs. before	−40 (−52 to −24)	< 0.001	−21 (−23 to −19)	< 0.001	0.76 (0.61 to 0.96)	0.02

*CI* Confidence interval

The rate ratio is the value representing the ratio of Shiga Prefecture’s percent change before and after intervention compared to that of Japan. Specifically, a rate ratio below 1 indicates a decline in the stillbirth rate of Shiga compared to the national average, while a ratio exceeding 1 indicates an elevation in Shiga’s stillbirth rate

### Change in the possibility of stillbirth prevention and the timing of stillbirth after the intervention

Table 4 shows that the rate of potentially preventable stillbirths in the late-intervention period was 13%, which was significantly lower than in the before-intervention period (31%, *p* < 0.001). Although the rate of potentially preventable stillbirths in the early-intervention period slightly decreased compared to the before-intervention period, the difference was not statistically significant (*p* = 0.43). The proportion of antepartum stillbirths, excluding intrapartum stillbirths, remained low and showed little change throughout each period (Table 4). Stillbirths at ≥ 28 weeks of gestation were more common than those at < 28 weeks, with no significant difference in this distribution between the before-intervention and post-intervention periods (early or late) (Table 4). The primary cause of stillbirth could not be determined in 43% of the cases. Among the cases with an identified cause, umbilical cord-related problems accounted for 18%, followed by placental abruption in 9% (data not shown).

**Table 4:** Possibility of stillbirth prevention and timing of fetal death in each period

	Before (2008-12)	Early (2013-17)	Late (2018-22)	*P* value	
				Before vs. Early	Before vs. Late
Analyzed	204	179	114		
Determinable	199	176	111		
Not determinable	5	3	3		
Preventability				0.43	< 0.001
Possible	62 (31)	48 (27)	14 (13)		
Impossible	137 (69)	128 (73)	97 (87)		
Timing of fetal death				0.21	0.78
Intrapartum	10 (5)	15 (8)	4 (4)		
Antepartum	194 (95)	164 (92)	110 (96)		
Gestational age at stillbirth				0.52	1.00
< 28 weeks	65 (32)	63 (35)	37 (32)		
≥ 28 weeks	139 (68)	116 (65)	77 (68)		

Data are shown as n (%)

## Discussion

We found that the stillbirth rate (≥ 22 weeks of gestation per 1,000 births) in Shiga Prefecture significantly decreased after the implementation of our intervention, in which we informed obstetricians, midwives, public health nurses, and pregnant women in our region about recommendations to prevent stillbirths. In addition, we found a significant reduction in possible preventable stillbirths after the intervention.

First, we found that the stillbirth rate in Shiga decreased significantly after the intervention. This population-based study in Japan discusses the association between disseminating recommendations to prevent stillbirth and the reduction in stillbirth rates in a region. A unique feature of these recommendations to prevent stillbirths, derived from a review of all stillbirth cases in Shiga Prefecture, is that they are directed to both obstetricians and pregnant women. We found several cases of high-risk pregnancies that might have resulted in stillbirths as a result of substandard management in primary obstetric clinics rather than in tertiary perinatal centers. Therefore, we informed obstetricians in primary clinics in Shiga of our recommendations for earlier referral of high-risk pregnancies, including monochorionic-diamniotic twins, fetal growth restriction, and hypertensive disorders of pregnancy, to higher- level perinatal centers. Although our recommendations are similarly described in obstetric guidelines [8] and the Japanese report for the prevention of cerebral palsy to prevent poor perinatal outcomes [9], these preventable cases suggest that the recommendations for improving poor perinatal outcomes have not been adequately shared with all obstetricians in our region. We speculate that the direct provision of these recommendations to obstetricians in our region may have led to improvements in perinatal management and, consequently, a reduction in regional stillbirths. Since 2013, no new tertiary perinatal centers specializing in high-risk pregnancies have been established in Shiga Prefecture. The consistency in the provision of advanced perinatal care during the intervention period suggests that factors other than our intervention are less likely to have contributed to stillbirth reduction in our region.

We also found that many intrauterine fetal death cases were diagnosed at the time of maternal visits to healthcare providers due to the perception of decreased fetal movements (DFM), and that the majority of these visits were excessively delayed after their perception of DFM [10]. Pregnant women were asked to count fetal movements (FM) and report to their care provider if it took much longer to perceive FM than usual [7]. A recent Cochrane review did not provide sufficient evidence to support the notion that the formal fetal movement count (FMC) [11] was beneficial for stillbirth prevention, and obstetric guidelines in Japan do not necessarily recommend FMC to prevent stillbirths [8]. However, as we found previously that excessively delayed maternal visits to a health care provider after the perception of DFM significantly decreased after the intervention, informing pregnant women about the FMC may have been associated with a reduction in the stillbirth rate in our region. As mentioned above, in addition to the recommendations in obstetric care to healthcare providers, the education of FMC for pregnant women may have significantly reduced the stillbirth rate in Shiga Prefecture, making it the second-lowest rate among prefectures in Japan after the intervention. Nevertheless, attention should also be paid to the potential for unintended consequences of FMC, such as increased maternal anxiety.

Secondly, we showed a significant reduction in possibly preventable stillbirths after the intervention period. This finding also suggests that the provision of recommendations for the prevention of stillbirths to healthcare providers and pregnant women could be effective in their prevention. There is no generally accepted definition for a preventable cause of stillbirth [12]. Page et al. classified stillbirths as potentially preventable, excluding fetuses younger than 24 weeks’ gestation, weighing < 500 g, and with major anomalies or genetic conditions [12]. Conversely, Sameshima reported that the preventability of perinatal death was determined by a majority of the participants at an audit conference [13]. In Japan, the survival to discharge rates for births before 24 weeks’ gestation are 36% at 22 weeks, 63% at 23 weeks, and 55% for infants with a birth weight below 500 g [14, 15]. In addition, in the current situation in Japan, where the survival rate of children with chromosomal abnormalities such as trisomy 18 surviving after intensive care is increasing [16–18], it is not necessarily appropriate to apply the criteria for preventable stillbirth, excluding major anomalies or genetic conditions according to Page et al. [12]. Our finding of a significant decrease in preventable stillbirth cases based on our criteria after the intervention suggests that the determination of preventable cases was appropriate and that the recommendations for stillbirth prevention were effective.

We have several limitations in the current study. First, we could not show that the reduction in stillbirth rates in our region was a direct outcome of the intervention. As it is extremely difficult to accurately identify the number of stillbirths prevented by awareness of these recommendations, we cannot clearly explain how our intervention contributed to a reduction in our regional stillbirths. However, data indicating a significant decrease in the proportion of stillbirths retrospectively judged to be avoidable following the intervention compared to before the intervention could suggest the impact of the intervention on reducing the stillbirth rate in Shiga. We did not identify any reports of similar interventions conducted in other regions of Japan. Therefore, we suggest there is an association between this intervention and a significant reduction in the stillbirth rate in our region. Next, decisions about the preventability of stillbirth are somewhat subjective and lack objectivity. There were no specific or objective definitions or criteria established for determining preventability, and decisions were made based on consensus among all reviewers. In making these judgments, the reviewers referred to the national obstetric guidelines available at the time as a reference. Even in instances where a consensus was not reached, the final decision was made based on the majority vote of the reviewers. Further research is needed to establish evidence-based guidelines for evaluating preventability and for assessing the effectiveness of interventions such as fetal movement counting in reducing stillbirths.

## Conclusion

We concluded that the stillbirth rate in Shiga Prefecture significantly decreased after intervention to inform healthcare providers and pregnant women in our region about recommendations to prevent stillbirths. Dissemination of our recommendations for stillbirth prevention may contribute to a reduction in stillbirths in other areas.

## Supplementary Information

Supplementary Material 1.

## Data Availability

The datasets analyzed in the current study on demographic trends are available in the repository of the Ministry of Health, Labour and Welfare (https://www.e-stat.go.jp/en). Data on the preventability of stillbirth is provided in the supplementary information files.

## Abbreviations

CI: Confidence interval
DFM: Decreased fetal movements
FM: Fetal movement
FMC: Fetal movement count
MHLW: Ministry of Health, Labour and Welfare
